# Supplementary material for: Infrared Evanescent Wave Sensing Based on a Ge10As30Se40Te20 Fiber for Alcohol Detection
Source: Sensors (Basel). 2023 May 17;23(10):4841. doi: 10.3390/s23104841 (PMC10220678; doi:10.3390/s23104841)
Supplement: Supplementary file 1 [file sensors-23-04841-s001.zip › sensors-2362728-supplementary.pdf]

Supporting information

Infrared Evanescent Wave Sensing Based on a $Ge_{10}As_{30}Se_{40}Te_{20}$ Fiber for Alcohol Detection

Zijian Li [1,2], Yongkun Zhao [1,2], Tianxiang You [1,2], Jihong Zhu [2,3], Mengling Xia [2], Ping Lu [1,2], Xianghua Zhang [1,4] and Yinsheng Xu [1,*]

1 State Key Laboratory of Silicate Materials for Architectures, Wuhan University of Technology, Wuhan 430070, China
2 School of Materials Science and Engineering, Wuhan University of Technology, Wuhan 430070, China
3 State Key Laboratory of Optical Fiber and Cable Manufacture Technology, Yangtze Optical Fibre and Cable Joint Stock Limited Company (YOFC), Wuhan 430073, China
4 Institut Des Sciences Chimiques de Rennes UMR 6226, Centre National de la Recherche Scientifique (CNRS), Université de Rennes 1, 35042 Rennes, France
* Correspondence: xuyinsheng@whut.edu.cn

As shown in Figure S1, the tapered fiber can be divided into three parts, untapered fiber, taper transition, and taper waist, whose lengths were 6 mm, 2 mm, and 30 mm, respectively. The diameter of untaper fiber was 380 μm and waist diameter (d_w) was 31 μm.

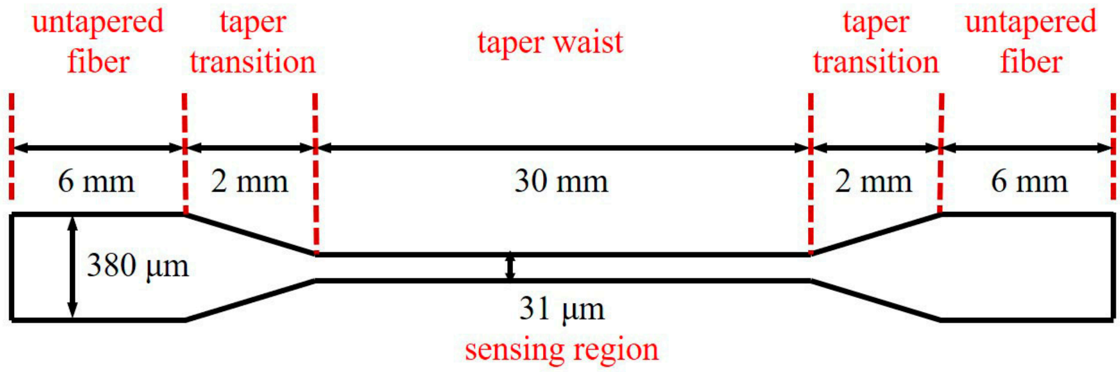

Figure S1. Geometry of the tapered fiber with d_w = 31 μm.
